# In Situ Synthesis of Gold Nanoparticles from Chitin Nanogels and Their Drug Release Response to Stimulation

**DOI:** 10.3390/polym16030390

**Published:** 2024-01-31

**Authors:** Jianwei Zhang, Wenjin Zhu, Jingyi Liang, Limei Li, Longhui Zheng, Xiaowen Shi, Chao Wang, Youming Dong, Cheng Li, Xiuhong Zhu

**Affiliations:** 1 College of Forestry, Henan Agricultural University, Zhengzhou 450002, China; zhangjianweiwhu@163.com (J.Z.); 15009315909@163.com (W.Z.); hnpy0110@126.com (L.L.); zhenglh@henau.edu.cn (L.Z.); lichengzzm@163.com (C.L.); 2 College of Landscape Architecture and Art, Henan Agricultural University, Zhengzhou 450002, China; l374811491@163.com; 3 School of Resource and Environmental Science, Wuhan University, Wuhan 430079, China; shixw@whu.edu.cn; 4 College of Materials Science and Technology, Nanjing Forestry University, Nanjing 210037, China; youming.dong@njfu.edu.cn

**Keywords:** chitin nanogels, au nanoparticles, photothermal effect, drug release

## Abstract

In this study, gold nanoparticles (AuNPs) were synthesized in situ using chitin nanogels (CNGs) as templates to prepare composites (CNGs@AuNPs) with good photothermal properties, wherein their drug release properties in response to stimulation by near-infrared (NIR) light were investigated. AuNPs with particle sizes ranging from 2.5 nm to 90 nm were prepared by varying the reaction temperature and chloroauric acid concentration. The photothermal effect of different materials was probed by near-infrared light. Under 1 mg/mL of chloroauric acid at 120 °C, the prepared CNGs@AuNPs could increase the temperature by 32 °C within 10 min at a power of 2 W/cm^2^. The Adriamycin hydrochloride (DOX) was loaded into the CNGs@AuNPs to investigate their release behaviors under different pH values, temperatures, and near-infrared light stimulations. The results showed that CNGs@AuNPs were pH- and temperature-responsive, suggesting that low pH and high temperature could promote drug release. In addition, NIR light stimulation accelerated the drug release. Cellular experiments confirmed the synergistic effect of DOX-loaded CNGs@AuNPs on chemotherapy and photothermal therapy under NIR radiation.

## 1. Introduction

Stimuli-responsive polymer systems are a highly promising area of research, especially in drug delivery. These polymers can be engineered to release drugs in a controlled and targeted manner, resulting in more effective treatment with fewer side effects. For instance, polymers that respond to changes in pH or temperature can be used to release drugs, specifically in areas where pH or temperature is altered, such as cancerous tumors. Thus, combining multiple therapies using stimulus-responsive drug delivery systems can be more effective in treating cancer [1]. At present, synergistic therapy, which combines chemotherapy and photothermal therapy, has attracted wide attention because of its simple operation, low cost, and remarkable effect [2,3,4]. There are two important components in synergy, namely, scaffold nanomaterials and photothermal conversion nanomaterials. For efficient delivery systems, the scaffold materials should be easy to synthesize, with a high loading rate, high encapsulation rate, and good biocompatibility [5,6], while photothermal conversion nanomaterials need to have high photothermal conversion efficiency [7]. Gold nanoparticles (AuNPs) currently have a wide range of applications in biomedical fields such as biosensing, cellular imaging, drug delivery, and cancer therapy due to the plasmon resonance absorption bands on their surfaces [8,9,10,11]. The position of the plasmon resonance absorption band depends on the size and shape of the nanoparticles, the surrounding environment, the aggregation state, and the distance between the particles [12,13,14]. Therefore, AuNPs can absorb incident energy in the near-infrared region and convert it into heat, which can damage the cell membrane of cancer cells when the temperature increases to a certain level, thus leading to the apoptosis of cancer cells [15]. Currently, researchers have developed a series of photothermal materials based on AuNPs that can be used for cancer therapy [16,17,18]. Hirsch et al. prepared NIR-absorbable silica/gold core-shell particles for photothermal destruction of human breast cancer cells and solid tumors [19]. Teng Cui et al. constructed thiol-capped polyethylene glycol (PEG) molecules to bind AuNPs via thiol-Au covalent bonds and loaded DOX by molecular design, and finally verified the synergistic effect of this composite on cancer cells via cellular experiments [20]. The plasmonic resonance effect of AuNPs depends largely on their shapes and sizes; the larger the particle size, the more obvious their plasmonic resonance effect. However, large-size AuNPs are destructive to the colloidal stability of nanoparticles and are very easy to aggregate and sink, which affects their function [21]. Therefore, it is crucial to prepare AuNPs that can exist stably in the system and have good photothermal effects.

**Figure 1:**
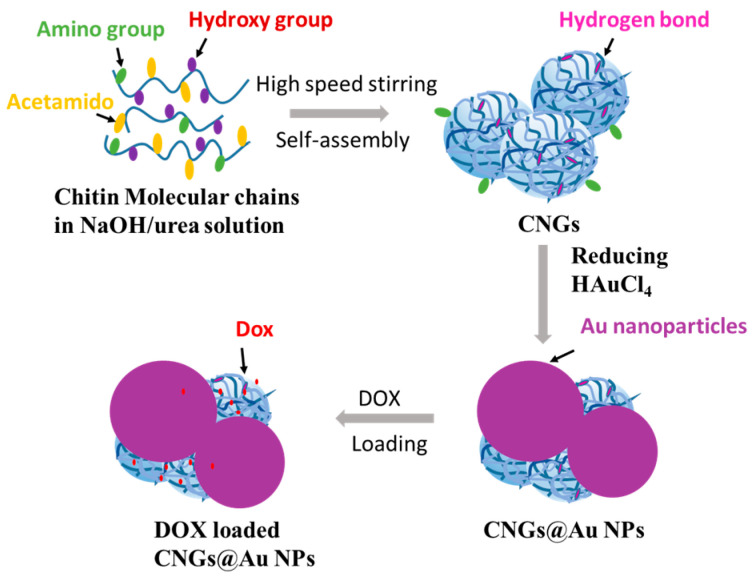
Preparation of DOX-loaded CNGs @AuNPs.

Chitin as a natural polymer has good biocompatibility and degradability, with a wide range of applications in drug release, wound dressings, tissue engineering, and other biomedical fields [22,23,24]. Since chitin contains a small amount of amino groups, it can reduce metal ions in order to prepare metal particles [25]. Therefore, we used the chitin nanogel prepared in our previous work [26] as a template to reduce chloroauric acid to generate AuNPs and immobilize them on the surface of chitin through the reducing effect of its amino group and its chelating effect (Figure 1). AuNPs with different sizes and morphologies have been applied in various fields, such as catalytic reactions [27] and electrochemical bio-detection [28]; however, they affect the catalytic and optical properties of AuNPs. Therefore, the size tuning of AuNPs is a crucial consideration in their synthesis. In this study, we prepared AuNPs with different sizes and shapes by varying the reaction conditions, such as the concentration of chloroauric acid and temperature during the chitin nanogel reaction. The chitin nanogel/AuNP composites (CNGs@AuNPs) exhibiting the most pronounced plasmon resonance effect were selected. Then DOX was loaded into CNGs@AuNPs to explore their release behavior under different pH values, temperatures, and near-infrared light stimulations. Finally, the effect of CNGs@AuNPs as DOX drug carriers on hepatocellular carcinoma (HepG2) cells under NIR radiation was investigated via in vitro cellular experiments. This system combined chemotherapy and photothermal therapy to play a common role and is expected to be applied in cancer therapy. 

## 2. Materials and Methods

### 2.1. Materials

Sodium hydroxide (NaOH), urea (CO(NH_2_)_2_), hydrochloric acid (HCl, 35%), and chloroauric acid (HAuCl_4_) were purchased from SINOPHARMA Group Chemical Reagent Co., Ltd. (Beijing, China). Adriamycin hydrochloride (DOX, 98%) was purchased from Wuhan Remote Co-creation Technology Co. (Wuhan, China). An industrial-grade flake chitin was purchased from Qingdao Yunzhou Biotechnology Co. (Qingdao, China), and its average molecular weight is 5 × 10^5^ Da. Dulbecco’s medium, penicillin, streptomycin, fetal bovine serum, and thiazolyl blue (MTT) were purchased from Yingjie Company (Shanghai, China).

### 2.2. Preparation of CNGs@AuNPs

Firstly, the raw material for chitin was ground into powder and then purified. Chitin powder was immersed in a 1 mol/L NaOH solution for 12~16 h at room temperature to remove protein and then washed to neutral using deionized water filtration. The raw material of chitin was then immersed in 5% hydrochloric acid for 10~12 h at room temperature to remove calcium carbonate and then washed to neutral using deionized water filtration. The alkaline immersion and acid immersion were performed twice. The chitin material was dispersed in a 0.3 wt% NaClO buffer solution, heated in an 80 °C oil bath for 3 h for decolorization, filtered and washed with deionized water to neutralize, dried, and sealed for storage. The deacetylation degrees of purified chitin and chitin in CNGs nanogels were measured using the potentiometric titration method [29]. CNGs were prepared by first dispersing 2 wt% purified chitin in 8 wt% NaOH/4 wt% urea, mixing thoroughly, and placing it at −25 °C for three cycles of freezing to obtain a transparent chitin solution. The 2% chitin solution was stirred at 10,000 r/min for 30 min in a high-speed disperser to obtain an emulsion solution. The chitin nanogel emulsion was dialyzed in distilled water by a dialysis bag with a molecular weight cut-off of 8000. In the process, the emulsion pH change was monitored. When the chitin nanogel was neutral, we continued dialysis for three days to ensure that the urea was completely dialyzed out.

Subsequently, gold nanoparticles were synthesized in 10 mg/mL of CNG suspension as follows: 10 mg/mL of CNG suspension was mixed with varying volumes of 10 mg/mL of chloroauric acid solution, resulting in final concentrations of 0.25 mg/mL, 0.5 mg/mL, 1 mg/mL, and 1.5 mg/mL of chloroauric acid. The reaction system was then continuously stirred with a magnetic stirrer at a constant temperature of 40 °C for 20 h. In addition, the reaction of chloroauric acid with CNG suspension at final concentrations of 0.5 mg/mL and 1 mg/mL was carried out at 65 °C, 90 °C, and 120 °C. The reaction rate accelerated, which significantly shortened the reaction time due to the increase in temperature. Therefore, the reaction time at 65 °C, 90 °C, and 120 °C was 4 h, 1 h, and 0.5 h, respectively. The reaction products prepared under different conditions were photographed and denoted CNGs@AuNPs.

### 2.3. Conformal Characterization of CNGs@AuNPs

The microscopic morphology of the prepared CNGs@AuNP samples was observed by high-resolution transmission electron microscopy (HRTEM, JEM-2100, Japan) [30,31]. The samples were dispersed and diluted to a suitable magnification with an ultrasonic cell disruptor (ultrasonic cell disrupt, SB-3200D, China). The suspension was then added dropwise on a copper grid and allowed to dry naturally for observation.

### 2.4. Absorption Spectroscopic Determination of CNGs@AuNPs

The 10 mg/mL CNGs@AuNPs prepared under different conditions were first frozen by 8 wt% NaOH/4 wt% urea cryogenic cycling to completely dissolve the chitin in the composites and avoid its interference with the AuNP test. The obtained suspension of AuNPs was then scanned on a UV-visible spectrophotometer (UV-Vis, UV-7000, Japan). The location of the maximum peak on the scanning curve was recorded [32].

### 2.5. Experiments on the Photothermal Properties of CNGs@AuNPs

A quantity of 1 mL of CNGs@AuNPs prepared under different conditions was added to cuvettes and placed 5 cm from the position directly opposite to the light source of the near-infrared laser (NIR, PSU-H-LED808, China) [33]. A thermocouple thermometer probe was inserted into the solution of the cuvette as close to the edge as possible to avoid errors in temperature measurements caused by direct NIR radiation. The initial temperature was recorded, and the operating current of the light source was adjusted to control the power of the NIR. A timer was then set, and the temperature of the solution was recorded at 1 min intervals. This experiment was repeated three times for each set of samples.

### 2.6. UV Standard Curve Plotting for DOX

A reservoir solution of 0.5 mg/mL was prepared by weighing 50 mg DOX with a high-precision balance and dissolving in 250 mL PBS. Quantities of 0 mL (blank), 0.1 mL, 0.2 mL, 0.5 mL, 1 mL, 2 mL, and 5 mL of DOX reservoir solution were pipetted accurately into a 50 mL volumetric flask and diluted with PBS to the scale. After configuring each gradient solution, the relative absorbances at 480 nm were recorded via a UV spectrophotometer. Finally, the UV curve was plotted to produce the standard curve equation of DOX.

### 2.7. CNGs@AuNP DOX Loading and DOX-Controlled Release Experiments

#### 2.7.1. DOX Adsorption Experiment

The pH of CNGs@AuNPs was adjusted to neutral with 0.1 M NaOH and HCl. A quantity of 20 mL of CNGs@AuNPs at 4.5 mg/mL was added to a 50 mL conical flask, 10 mL of 200 mg/L Adriamycin hydrochloride (DOX) was added, and the mixture was constantly oscillated at 60 r/min at 25 °C for 24 h. The loaded samples were centrifuged, and the absorbance of the supernatant was tested to determine the amount of unloaded DOX using the DOX standard curve. The amount of DOX loaded in the CNGs@AuNP composite was then calculated.

The doxorubicin loading capacity of the samples was calculated by measuring the concentration of doxorubicin before and after adsorption, which was quantitated by monitoring the absorbance of doxorubicin at 480 nm with a UV spectrophotometer. The drug loading capacity (D, mg/g) was estimated according to the following equation:

$$D=\frac{M_{0}}{M_{0}+M_{1}}$$

where *M*_0_ is the quantity of doxorubicin (mg) contained in the CNGs@AuNP composite and *M*_1_ is the quantity of CNGs@AuNP composite (g).

#### 2.7.2. In Vitro Drug Release Experiments under Different pH Conditions

A quantity of 1 mL of CNGs@AuNPs loaded with DOX was added to 9 mL of buffer with pH 4.5, 6.0, and 7.4. At predetermined time points and a constant temperature of 37 °C, 200 µL of supernatant was taken and replenished with 200 µL of fresh buffer, keeping the total volume constant. The absorbance of the supernatant was measured at 480 nm to determine the concentration of DOX released. The time–DOX cumulative release curve was then plotted.

#### 2.7.3. In Vitro Drug Release Experiments at Different Temperatures

A quantity of 1 mL of CNGs@AuNPs loaded with DOX was added to 9 mL of buffer with pH 7.4, and place at 25 °C and 40 °C. At predetermined time points, 200 µL of supernatant was taken and replenished with 200 µL of fresh buffer, keeping the total volume constant. The absorbance of the supernatant was measured at 480 nm to determine the concentration of DOX released. The time–DOX cumulative release curve was then plotted.

#### 2.7.4. Experiment under NIR Laser Stimulation

A quantity of 1 mL of CNGs@AuNPs loaded with DOX was added to 9 mL of buffer with a pH of 4.5 at 37 °C. At predetermined time points, 200 µL of supernatant was added to a 96-well plate and replenished with 200 µL of fresh buffer, keeping the total volume unchanged. In this process, the solution was irradiated with a NIR laser of 2 W/cm^2^ and 808 nm for 15 min at 1 h, 2 h, and 3 h of drug release. Similarly, 200 µL of supernatant was added to a 96-well plate and replenished with 200 µL of fresh buffer after 15 min of irradiation each time. The absorbance of the supernatant was measured at 480 nm to determine the concentration of DOX released. The time–DOX cumulative release curve was then plotted.

### 2.8. In Vitro Photothermal Therapy and Photothermal Therapy/Chemotherapy Cell Experiments

Antitumor activity of photothermal therapy and combined in vitro photothermal therapy/chemotherapy using MTT assay of selected hepatocellular carcinoma cells (HepG2) study samples [34,35] was carried out. The HepG2 cells were provided by the Department of Medicine in Wuhan University. Four experimental groups were set: CNGS@AuNPs, CNGS @AuNPs + NIR, CNGS@AuNPs@DOX, and CNGS@AuNPs@DOX + NIR. In addition, five concentration gradients were established: 0 µg/mL, 50 µg/mL, 100 µg/mL, and 250 µg/mL. Among these, the final release equilibrium concentration in four concentration gradients of CNGS@AuNPs@DOX and CNGS@AuNPs@DOX + NIR groups was 0 µg/mL, 3.75 µg/mL, 7 µg/mL, and 15 µg/mL. After the cells were co-cultured with the material for 24 h, the NIR-irradiated group was irradiated with an 808 nm laser at a power of 5 w/cm^2^ for 5 min per well. The cells were returned to the incubator for another 24 h of co-incubation. Next, 10 µL of 5 mg/mL MTT was added to each of the cell plate wells of the above cultures and incubated for 4 h. Subsequently, the original culture medium was removed, rinsed twice with PBS, and then DMSO (100 µL/well) was added to each well and shaken for 10–15 min. Finally, the OD value was measured at the wavelength of 490 nm, whereby the OD value was proportional to the number of cells. The effect of the material on the cell proliferation ability, which is the cell survival rate, was evaluated. The cell survival rate is expressed in the following formula:

$$\mathrm{Cell}\;\mathrm{viability}\;(\%)=\frac{\left(OD\;value\;for\;each\;concentration\;group-0\;well\;OD\;value\right)}{\left(Negative\;control\;OD\;value-0\;well\;OD\;value\right)}\times 100\%$$

## 3. Results and Discussion

### 3.1. Effect of Chloroauric Acid Concentration on the Morphology of CNGs@AuNPs

**Figure 2:**
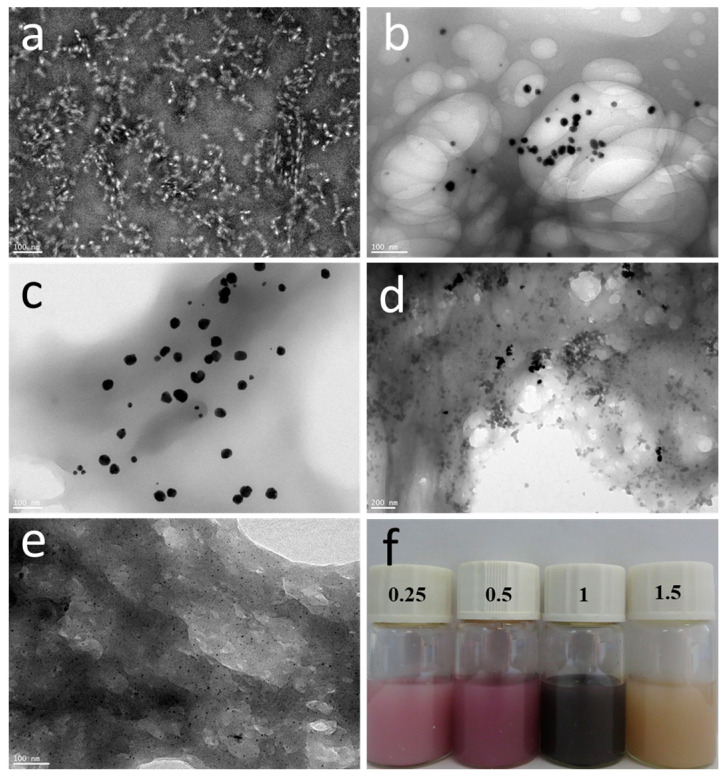
TEM images of chitin nanogels (**a**); TEM images and photograph of AuNPs produced with different HAuCl_4_ concentrations at 40 °C: (**b**) 0.25 mg/mL, (**c**) 0.5 mg/mL, (**d**) 1 mg/mL, (**e**) 1.5 mg/mL, and (**f**) photograph.

The chitin nanogels were prepared based on our previous method [26]. The chitin nanogel dispersion was stained with phosphotungstic acid and observed via transmission electron microscopy, as shown in Figure 2a. The nanogels are circular-like particles with particle sizes between 20 and 30 nm, and most of them are clustered. The experimental results showed that the degree of deacetylation of purified chitin and chitin nanogel is about 16% and 28%, respectively. Chloroauric acid is reduced to AuNPs by the amino reduction in chitin, whereas AuNPs are well-supported and protected by chelation, which immobilizes AuNPs on their surface [36]. The microscopic morphologies of the prepared CNGs@AuNPs were observed by high-resolution transmission electron microscopy, which varied with different chloroauric acid concentrations. As shown in Figure 2, AuNPs generated by the in situ reduction of amino groups on chitin were attached to the chitin nanogel material to form a stable suspension and displayed different colors (Figure 2e). Most of the AuNPs obtained with the different chloroauric acid concentration gradients were relatively homogeneous and circular, with a few showing irregular polygonal shapes due to the inhomogeneity of some regions of the chitin nanogel.

**Figure 3:**
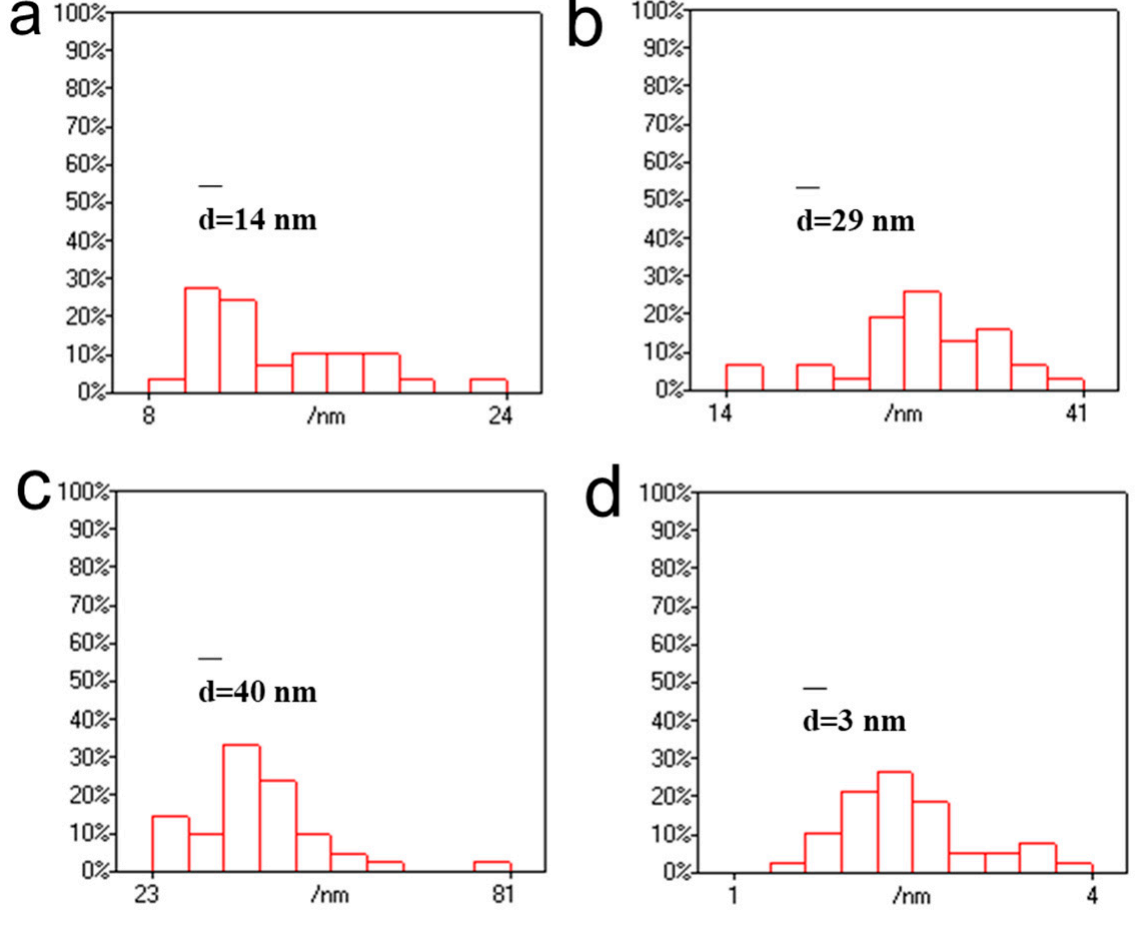
Statistical size distributions of AuNPs produced with different concentrations of HAuCl_4_ at 40 °C: (**a**) 0.25 mg/mL, (**b**) 0.5 mg/mL, (**c**) 1 mg/mL, and (**d**) 1.5 mg/mL.

AuNPs prepared at 40 °C with a concentration of 1 mg/mL of chloroauric acid had more aggregates, resulting in a darker cyan-blue color. The particle size of AuNPs was then statistically analyzed using Nanomeasure 1.2 software. As shown in Figure 3, the particle sizes of AuNPs produced at 40 °C with different chloroauric acid concentrations of 0.25 mg/mL, 0.5 mg/mL, 1 mg/mL, and 1.5 mg/mL were distributed in the ranges of 8–24 nm, 14–41 nm, 23–81 nm, and 1–4 nm, with an average particle size of 14 nm, 29 nm, 40 nm, and 3 nm, respectively. At a reaction temperature of 40 °C, the particle size of AuNPs increased with a gradual increase in concentration when the concentration of the chloroauric acid was below 1 mg/mL. However, when the chloroauric acid concentration was 1.5 mg/mL, the particle size of AuNPs was only 3 nm. This small-sized gold nanoparticle has received much attention in recent years in the fields of chemical catalysis, bioimaging, etc. [37,38,39]. In this experiment, we set the concentration of CNGs to 10 mg/mL, at which CNGs are more stable and do not aggregate or settle. Therefore, the concentration of chloroauric acid has a significant influence on the preparation of AuNPs. When the temperature was 40 °C, the size of the produced AuNPs increased with the increase in HAuCl_4_ concentration; however, the particle size of AuNPs was significantly reduced to 3 nm at 1.5 mg/mL of HAuCl_4_. At too low a concentration, the weak reducing property and the limited growth of crystals prevent the nanocrystals from reaching the saturation state of the system at the early stage of formation. There is also a possibility that when the concentration of chloroauric acid is low, the chitin molecular chains occupy the relative space for crystal growth, thus reducing the crystallinity of the crystals and hindering their growth. The formation of nanoparticles consists of two processes: the nucleation process of the crystals and the crystal growth. The nucleation process of the crystals is very rapid, while the crystal growth process is very slow [40]. In the concentration range of 0.25–1.0 mg/mL of HAuCl_4_, as the concentration of chloroauric acid increases, the particle size of the generated gold nanoparticles becomes larger. The reason for this is that after nucleation, with the further reduction of chloroauric acid, the Au crystal nuclei become larger and larger. An excessive concentration of HAuCl_4_ causes excessive nuclei to be formed at the initial stage in solution, forming nanoparticles dominated by fine particles. When the concentration of HAuCl_4_ was 1.5 mg/mL, too many nuclei were formed in the initial stage, and the consumption of amino was also relatively high. A large quantity of precursors and reducing agents was consumed in the nucleation stage; as a result, the growth rate of seeds was decelerated, which limited the size of the generated AuNPs [41]. 

### 3.2. Effect of Reaction Temperature on the Morphology of CNGs@AuNPs

**Figure 4:**
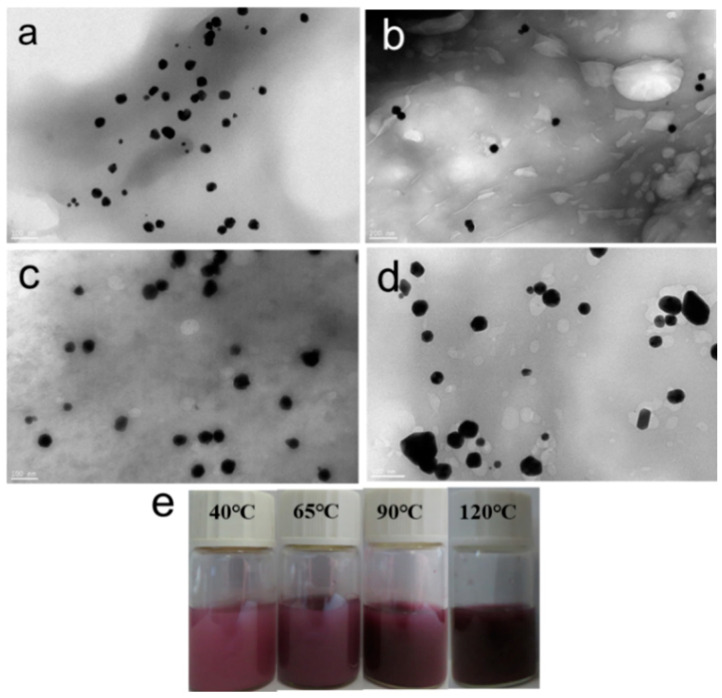
TEM images and a photograph of AuNPs produced with HAuCl_4_ concentration at 0.5 mg/mL under different temperatures: (**a**) 40 °C; (**b**) 65 °C, (**c**) 90 °C, (**d**) 120 °C, and (**e**) photograph.

**Figure 5:**
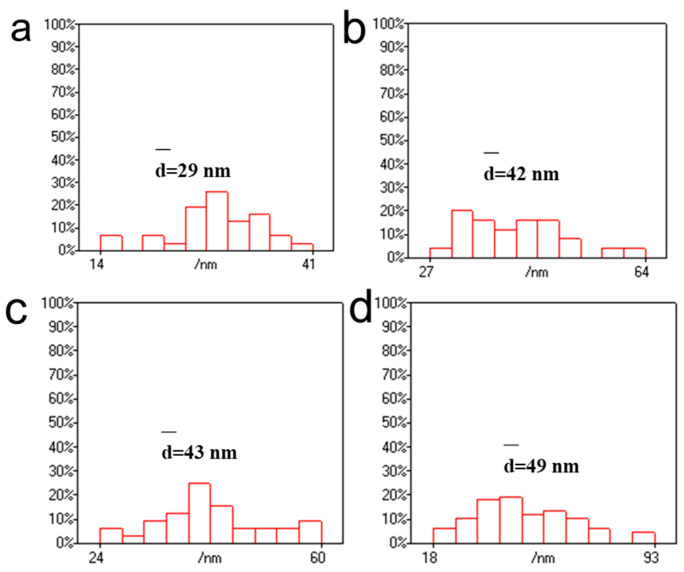
Statistical size distributions of AuNPs produced under different temperatures at HAuCl_4_ concentration of 0.5 mg/mL: (**a**) 40 °C, (**b**) 65 °C, (**c**) 90 °C, and (**d**) 120 °C.

The particle size of AuNPs in CNGs@AuNPs was not only related to the reaction concentration of chloroauric acid, but the reaction temperature also affected the synthesis of AuNPs. Four temperature gradients, 40 °C, 65 °C, 90 °C, and 120 °C, were selected, and the prepared CNGs@AuNPs at a chloroauric acid concentration of 0.5 mg/mL were observed via high-resolution transmission electron microscopy, as shown in Figure 4. Most of the prepared AuNPs were regular monodispersed spherical shapes. When the temperature was 120 °C, a small portion of the AuNPs were polygonal with larger particles and showed a darker purple color on the surface. Figure 5 shows the particle size distribution of AuNPs in the samples under different reaction temperatures. The AuNPs’ size ranges at 40 °C, 65 °C, 90 °C, and 120 °C were mainly distributed in 14–41 nm, 27–64 nm, 24–60 nm, and 18–93 nm, with an average particle size of 29 nm, 42 nm, 43 nm, and 49 nm, respectively. Therefore, the particle size of AuNPs increased with an increase in reaction temperature at 0.5 mg/mL chloroauric acid concentration. This is because the crystal growth was limited by its weak reducing properties at low temperatures; thus, the growth of Au nanocrystals was not saturated, and increasing the temperature led to further crystal growth.

**Figure 6:**
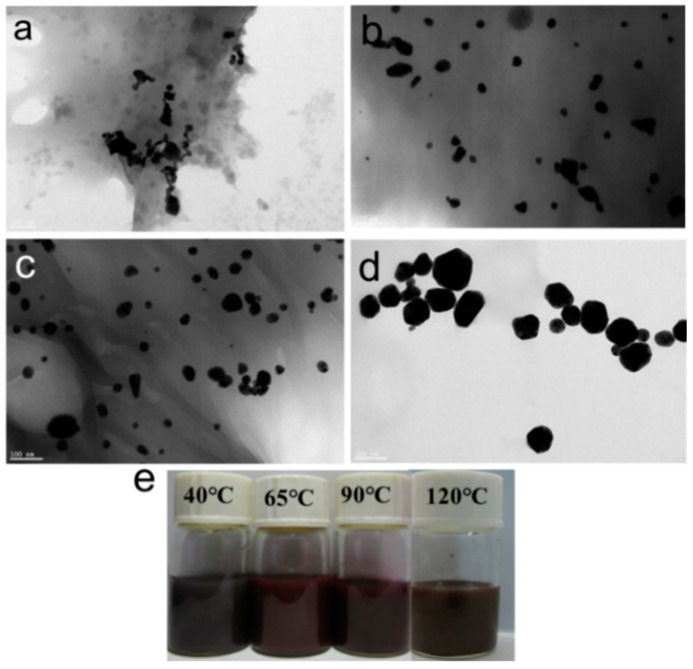
TEM images and a photograph of AuNPs produced under different temperatures at HAuCl_4_ concentration of 1 mg/mL: (**a**) 40 °C, (**b**) 65 °C, (**c**) 90 °C, (**d**) 120 °C, and (**e**) photograph.

**Figure 7:**
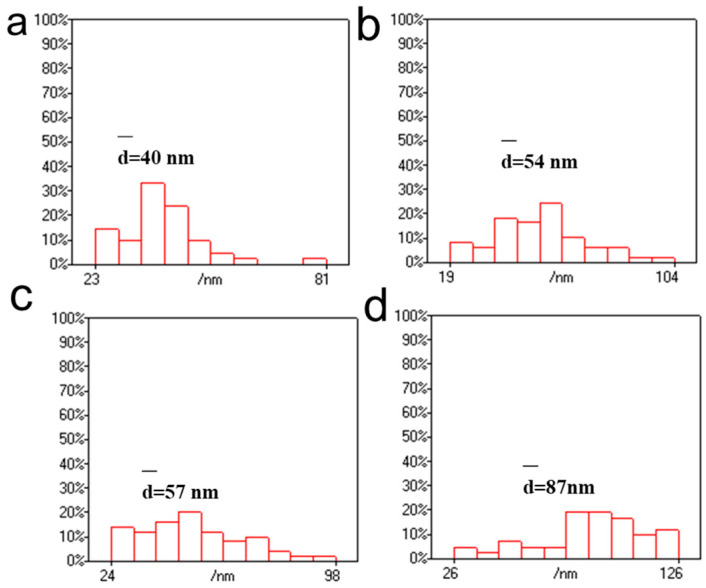
Statistical size distributions of AuNPs produced under different temperatures at HAuCl_4_ concentration of 1 mg/mL: (**a**) 40 °C, (**b**) 65 °C, (**c**) 90 °C, and (**d**) 120 °C.

In addition, the morphology of CNGs@AuNPs generated at 1 mg/mL chloroauric acid concentration under different reaction temperatures (40 °C, 65 °C, 90 °C, and 120 °C) is shown in Figure 6. It can be seen that the AuNPs generated at a chloroauric acid concentration of 1 mg/mL and 40 °C were partially aggregated and irregular. The AuNPs prepared at 65 °C and 90 °C were not uniform in size, with some exhibiting triangular shapes, whereas at 120 °C, most of them were polygonal in shape and relatively more homogeneous in size. Figure 7 showed that at a chloroauric acid concentration of 1 mg/mL, the particle sizes of AuNPs at 40 °C, 65 °C, 90 °C, and 120 °C were distributed in the ranges of 23–81 nm, 19–104 nm, 24–60 nm, and 18–98 nm, with their average particle sizes of 40 nm, 54 nm, 57 nm, and 87 nm, respectively. This trend was basically consistent with the chloroauric acid reaction concentration of 0.5 mg/mL. On the whole, the main factors affecting the size of AuNPs were the concentration and reaction temperature of HAuCl_4_. When the concentration of HAuCl_4_ is between 0.25 and 1 mg/mL, the size of the AuNPs increased with the increase in the concentration. However, when the concentration of HAuCl_4_ was 1.5 mg/mL, the size of the nanoparticles decreased. At the same time, the effect of temperature on the size of AuNPs was that the higher the temperature, the larger the size of AuNPs.

### 3.3. Absorption Spectral Analysis of CNGs@AuNPs

AuNP absorption depicts surface plasmon resonance absorption, which is closely related to the motion of free electrons on the metal surface [42]. When an electromagnetic wave acts on the surface of AuNPs, the surface plasma oscillates, and resonance occurs when the frequency of the electromagnetic wave is the same as the frequency of the plasma oscillation [43,44]. At the macroscopic level, this resonance manifests itself in the absorption of light by AuNPs.

**Figure 8:**
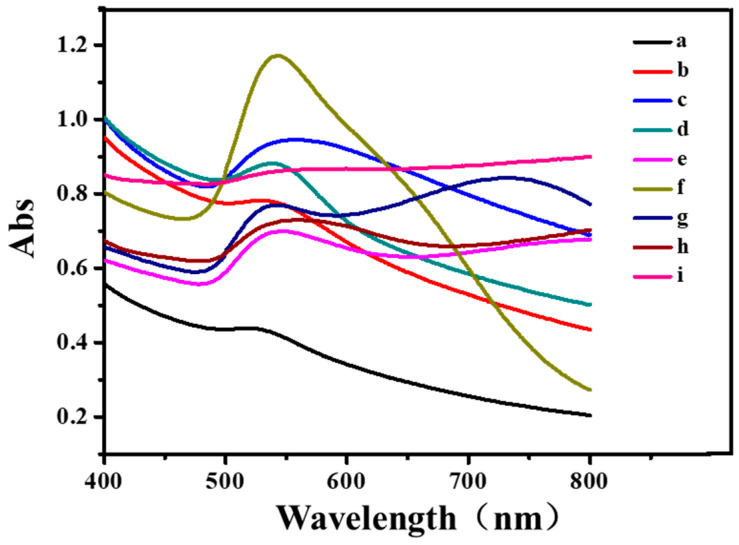
UV–Vis extinction spectra of different samples. AuNPs produced with different HAuCl_4_ concentrations at 40 °C: 0.25 mg/mL (**a**), 0.5 mg/mL (**b**), 1 mg/mL (**c**); AuNPs produced with different HAuCl_4_ concentrations at 65 °C: 0.5 mg/mL (**d**), 1 mg/mL (**e**); AuNPs produced with different HAuCl_4_ concentrations at 90 °C: 0.5 mg/mL (**f**), 1 mg/mL (**g**); AuNPs produced with different HAuCl_4_ concentrations at 120 °C: 0.5 mg/mL (**h**), 1 mg/mL (**i**).

Figure 8 shows the visible extinction spectra of the obtained CNGs@AuNPs prepared under different conditions. The chitin in the samples was dissolved completely before the scanning; hence, the scans were essentially visible extinction spectra of AuNPs. When the chloroauric acid concentrations were 0.25 mg/mL and 0.5 mg/mL, the spectra of AuNPs obtained by different temperature preparations had absorption peaks below 600 nm, which was weaker than the near-infrared band of 600–800 nm. Therefore, the photothermal conversion effect was not obvious when the chloroauric acid concentration was 0.25 mg/mL or 0.5 mg/mL. At 1 mg/mL chloroauric acid concentration, the spectral extinction peaks of AuNPs prepared at 40 °C and 65 °C were 556 nm and 547 nm, respectively, and the intensity of the infrared band at 600–800 nm was higher than that of the sample at a concentration of 0.5 mg/mL. The infrared band extinction of AuNPs prepared at 90 °C and 120 °C was significantly enhanced at 600–800 nm. It can be seen that the extinction spectra of AuNPs prepared with 1 mg/mL chloroauric acid concentration were all more intense in the near-infrared region than those of the products with a 0.5 mg/mL chloroauric acid concentration under the same condition. Additionally, the sizes of the AuNPs increased with an increase in temperature, which led to the gradual red-shift of the characteristic extinction peaks of the plasmon resonance.

By comparing the results of visible extinction spectroscopy with transmission electron microscopy and particle size analysis, it could be seen that the narrower the extinction peaks of AuNPs, the narrower the corresponding particle size distribution. With an increase in the particle size of AuNPs, the plasmonic resonance extinction peaks on the surface of AuNPs became increasingly broad. In addition, the extinction peaks were red-shifted with increasing particle size. 

### 3.4. Analysis of Photothermal Properties of CNGs@AuNPs

The use of AuNPs combined with anti-cancer drugs for cancer treatment is mainly based on the photothermal effect of AuNPs. By applying the photothermal material to the cancer site, the temperature increase under the stimulation of near-infrared light can kill the cancer cells. On the other hand, it can also stimulate the release of drugs from the material, playing a joint role in chemotherapy and photothermal therapy. Therefore, an evaluation of the photothermal effect of the drug carrier CNGs@AuNPs is needed prior to the controlled drug release investigation, with the aim of exploring whether CNGs@AuNPs can generate higher temperatures under the stimulation of an NIR light source for a short period of time, which in turn induces the temperature-responsive behavior of the polymer layer and realizes the NIR light-stimulated release of the drug.

**Figure 9:**
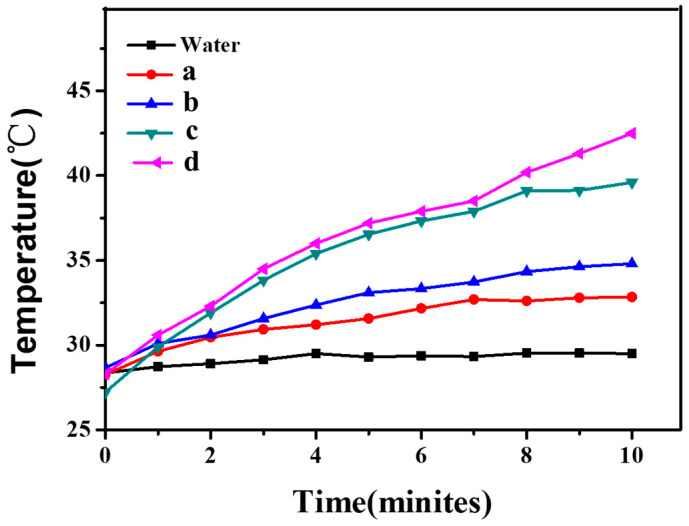
Photothermal effect of CNGs@AuNP samples prepared at HAuCl_4_ concentration of 0.5 mg/mL under different temperatures: 40 °C (**a**), 65 °C (**b**), 90 °C (**c**), 120 °C (**d**).

**Figure 10:**
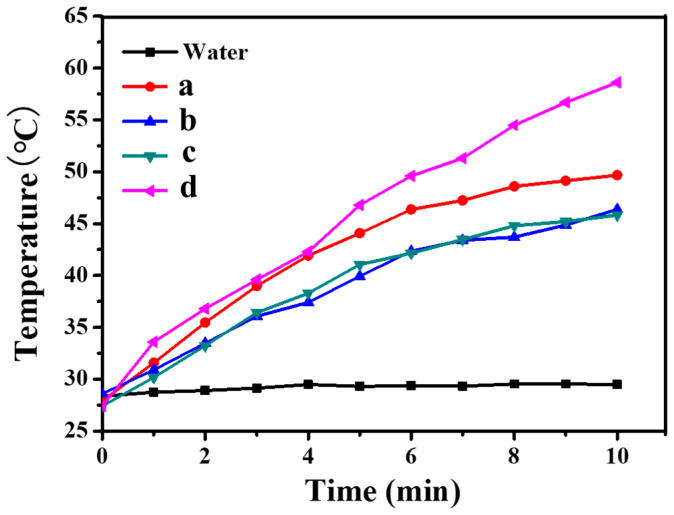
Photothermal effect of CNGs@AuNP samples prepared at HAuCl_4_ concentration of 1 mg/mL under different temperatures: 40 °C (**a**), 65 °C (**b**), 90 °C (**c**), 120 °C (**d**).

Figure 9 and Figure 10 show the photothermal conversion results of CNGs@AuNPs prepared at different temperatures with chloroauric acid concentrations of 0.5 mg/mL and 1 mg/mL, respectively. The materials were irradiated by an 808 nm laser at 2 W/cm^2^ for 10 min, and the temperature changes were observed. It was found that under the same chloroauric acid concentration, the higher the reaction temperature of the prepared AuNPs, the more rapid the increase in its temperature under NIR. Meanwhile, the photothermal effect of the AuNPs became more evident with increasing chloroauric acid concentration. At 120 °C, the temperature of CNGs@AuNPs prepared at 0.5 mg/mL chloroauric acid concentration increased by 15 °C in 10 min, from 28 °C to about 43 °C (Figure 9). The photothermal effect of CNGs@AuNPs prepared at 120 °C with a chloroauric acid concentration of 1 mg/mL was the best among all the samples, and the temperature increased by 32 °C from 28 °C to 60 °C (Figure 10). The photothermal effect was consistent with the visible spectrum and AuNP particle size analysis, i.e., the larger the particle size of the AuNPs, the more significant the photothermal effect. It also proved that the drug-carrying system had a strong photothermal conversion ability. When AuNPs and incident light waves interact, surface plasma resonance will occur. There are nanoparticles of very small size, and under the action of the electric field, the whole particle of the electron gas clusters will produce a displacement relative to the nanoparticle’s original position. With the fluctuation of the electromagnetic field and the oscillation, which causes the resonance of the internal crystal lattice, a large amount of thermal energy, i.e., AuNPs of photothermal effect, will be produced. Different sizes and shapes of gold nanostructures have different photothermal properties. For larger nanoparticles (greater than about 20 nm in the case of gold), the plasma resonance depends explicitly on the particle size, with the plasma band red-shifting as the particle size increases. Thus, for AuNPs, the larger the size, the higher the photothermal conversion efficiency [45]. 

### 3.5. CNGs@AuNPs Drug Loading and Stimulated Release

**Figure 11:**
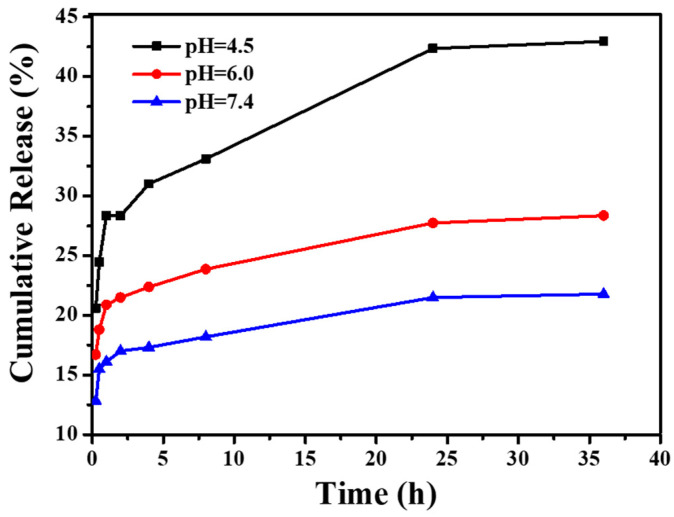
Effect of pH on the cumulative release profiles of DOX from CNGs@AuNPs.

A water-soluble anti-tumor drug, DOX, was chosen as a model, and the drug loading capacity of CNGs@AuNPs was 21.8 mg/g. The pH of the cellular microenvironment in organisms varies, with most somatic cells usually in the range of 7.35 to 7.45. Tumor cells are usually weakly acidic due to their vigorous metabolism and tissue hypoxia. Therefore, it is necessary to explore the effect of pH on the controlled release of DOX from CNGs@AuNPs. Figure 11 demonstrates the DOX release from CNGs@AuNPs at 37 °C under different pH conditions. The release of DOX was slow at pH 7.4, but it gradually accelerated as the pH decreased. The presence of amino groups in chitin nanogels caused their surface to protonate with more positive charges at low pH. This property helped release the positively charged DOX faster, which could be beneficial for the release of antitumor drugs.

**Figure 12:**
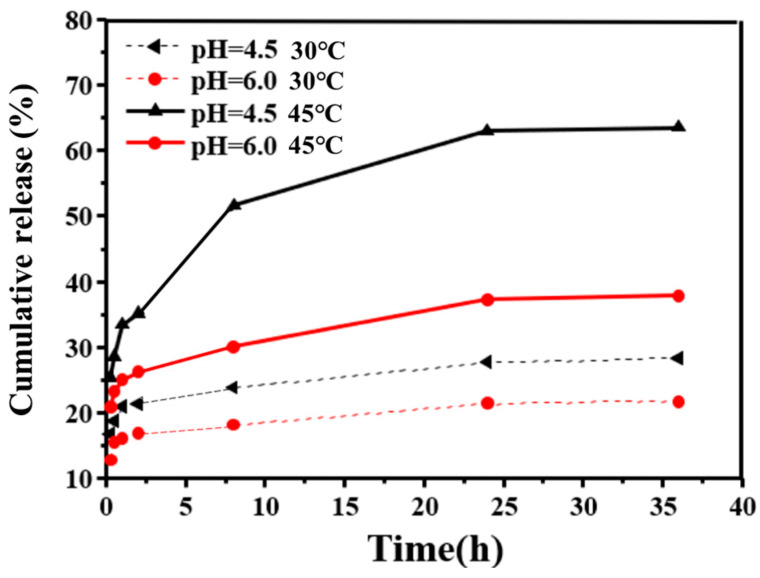
Effect of temperature on the cumulative release profiles of DOX from CNGs@AuNPs.

The photothermal effect of AuNPs not only kills cancer cells but also significantly affects the release of DOX from CNGs@AuNPs. Therefore, we explored the release of DOX from CNGs@AuNPs at pH 4.5 and 6.0 under temperatures of 30 °C and 45 °C, respectively. Figure 12 shows the effect of temperature on the cumulative release profiles of DOX from CNGs@AuNPs. The release of DOX from CNGs@AuNPs accelerated under elevated temperatures. Under the condition of 45 °C and pH 4.5, the cumulative DOX release rate was 65% at 36 h, while it was only 25% at 36 h under the condition of 30 °C and pH 4.5. This result lays the foundation for the photothermally controlled release of AuNPs. Since CNGs contain amino groups, they are protonated when the external pH is acidic, which causes a change in the electrostatic repulsion between functional groups. This leads to a greater solubility of the hydrogel, which in turn promotes the release of the drug from the system [46]. When the temperature increases, the release of the drug can be facilitated by the acceleration of the molecular Brownian farsightedness or solvation of the polymers [47]. 

**Figure 13:**
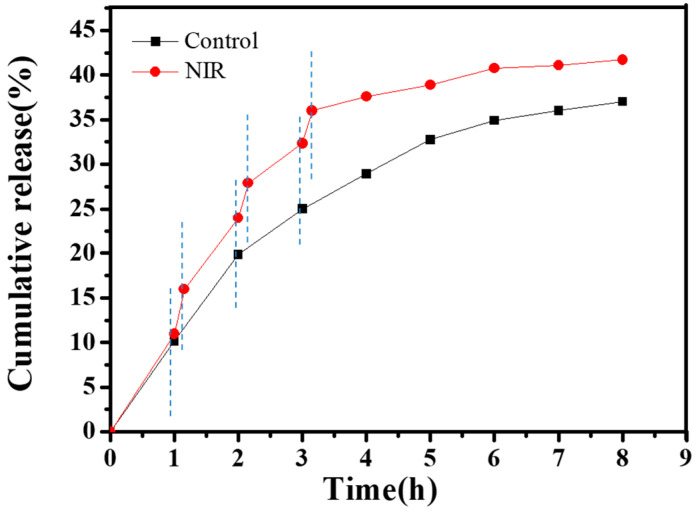
Effect of pulsed NIR irradiation on the release behavior of DOX-loaded CNGs@AuNPs obtained at HAuCl_4_ concentration of 1.0 mg/mL under 120 °C.

Finally, we investigated the effect of photo-responsiveness of CNGs@AuNPs obtained at a HAuCl_4_ concentration of 1.0 mg/mL at 120 °C on drug release by NIR radiation warming, as shown in Figure 13. The release medium was PBS buffer at pH 4.5. The black curve is the normal release group, which shows a cumulative DOX release rate of 37% in 8 h. CNGs@AuNPs were irradiated with 2 W/cm^2^ NIR light for 15 min at intervals of 1 h. The cumulative DOX release curves (red curve) obviously accelerated after radiation, obtaining a release rate of 42% at 8 h compared to 36% for the control group. The cumulative release rate of the control group was 36% at 8 h, and could reach 42% after NIR irradiation. As a kind of ambient temperature-sensitive drug-carrying system, CNGs@AuNPs could improve the antitumor effect of the drug by accelerating the drug release with an auxiliary NIR radiation.

### 3.6. In Vitro Photothermal Therapy and Photothermal Therapy/Chemotherapy Cell Experiments

**Figure 14:**
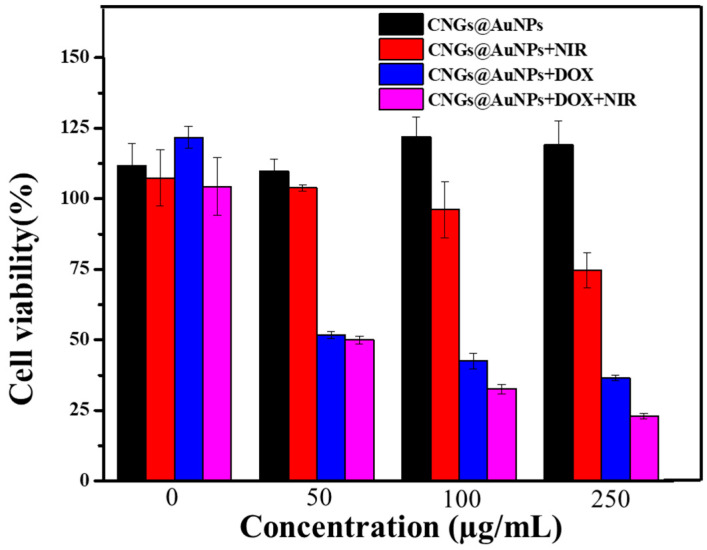
Relative viabilities of HepG2 cells cultured with different concentrations of CNGs@AuNPs and DOX-loaded CNGs@AuNPs with or without 808 nm laser irradiation (5.0 W/cm^2^ for 5 min).

Finally, we investigated the inhibitory effects of CNGs@AuNPs and DOX-loaded CNGs@AuNPs on HepG2 cells with or without NIR radiation, as shown in Figure 14. The cell survival rates of different concentrations of CNGs@AuNPs with HepG2 cells were above 100% after 48 h of co-culture, indicating that CNGs@AuNPs alone have good biocompatibility in the absence of NIR radiation. After 5 min of 808 nm NIR radiation, the apoptosis rate gradually increased with the increasing concentration of CNGs@AuNPs. At a concentration of 250 µg/mL, the survival rate of HepG2 cells was 74.7%. This is because a high concentration of CNGs@AuNPs has a more significant photothermal effect, and the increase in temperature will cause the destruction of DNA and proteins in the nucleus of the cells, resulting in cell apoptosis. DOX-loaded CNGs@AuNPs showed good chemotherapeutic effects on cells without NIR radiation, and the cell survival rates of samples with concentrations of 50 µg/mL, 100 µg/mL, and 250 µg/mL were 51.7%, 42.5%, and 36.5%, respectively. After 5 min of NIR radiation, the temperature of the DOX-loaded CNGs@AuNP system with different concentrations increased accordingly. The increase in temperature led to apoptosis and also accelerated the rate of DOX release. Therefore, under the dual action of DOX chemotherapy and photohyperthermia, the cell survival rates of 50 µ g/mL, 100 µ g/mL, and 250 µ g/mL DOX-loaded CNGs@AuNP samples were reduced to 49.9%, 32.6%, and 22.9%, respectively. In summary, DOX-loaded CNGs@AuNPs under NIR radiation on HepG2 hepatocellular carcinoma cells can exert a combined co-effect of chemotherapy and photothermal therapy in vitro.

## 4. Conclusions

In this paper, CNGs@AuNPs were prepared via in situ reduction of chloroauric acid using chitin nanogels as nanoscaffolds, and the chelating effect of chitin immobilized the AuNPs on their surfaces to form a stable suspension. The particle size of AuNPs was regulated by varying the reaction temperature and chloroauric acid concentration, with average particle sizes ranging from 3 nm to 89 nm. The photothermal effect of the materials was explored using NIR light, in which the CNGs@AuNPs prepared at 120 °C with a chloroauric acid concentration of 1 mg/mL exhibited good photo-responsiveness. In addition, its temperature can be increased by 32 °C in 10 min under NIR radiation of 2 W/cm^2^. The release of the anticancer drug DOX from CNGs@AuNPs was pH- and temperature-responsive, with low pH and high temperatures accelerating its release. In addition, the release rate of DOX could be accelerated by NIR radiation. Cellular experiments revealed that CNGs@AuNPs had a combined effect of chemotherapy and photothermal therapy on cancer cells under NIR radiation. In summary, the pH and NIR dual stimulation response behaviors of the controlled release of DOX by CNGs@AuNPs can be combined with chemotherapy and photothermal therapy, which is expected to be applied in the treatment of tumors.

## Data Availability

Data are contained within the article.
